# Identification and analysis of diverse cell death patterns in osteomyelitis via microarray-based transcriptome profiling and clinical data

**DOI:** 10.3389/fimmu.2025.1630172

**Published:** 2025-09-19

**Authors:** Tianxuan Feng, Peisheng Chen, Fengfei Lin

**Affiliations:** ^1^ Fujian University of Traditional Chinese Medicine, Fuzhou, Fujian, China; ^2^ Department of Orthopedic, Fuzhou Second General Hospital, School of Clinical Medicine of Fujian Medical University, Fujian Provincial Clinical Medical Research Center for First Aid and Rehabilitation in Orthopedic Trauma, Fuzhou, Fujian, China

**Keywords:** osteomyelitis, programmed cell death, transcriptome profiling, biomarkers, machine learning

## Abstract

**Background:**

Osteomyelitis (OM) is a debilitating infectious disease characterized by inflammation of the bone and bone marrow. Emerging evidence suggests that multiple forms of programmed cell death (PCD) contribute to its pathogenesis. However, the specific roles and interactions of these PCD types in OM remain largely undefined.

**Methods:**

Microarray-based transcriptome datasets related to OM were retrieved from the Gene Expression Omnibus (GEO) database. Thirteen PCD modalities were defined from the literature and specialized databases, including classical forms (e.g., apoptosis, autophagy) and non-classical forms (e.g., cuproptosis, entosis, ferroptosis). Gene Set Variation Analysis (GSVA) was used to evaluate pathway activities in OM, and their associations with immune infiltration, inflammation-related gene expression, and diagnostic value were systematically assessed. Weighted gene co-expression network analysis (WGCNA) was performed to identify essential modules and hub genes. A diagnostic model was constructed using machine learning with SHapley Additive exPlanations (SHAP), and candidate genes were validated in clinical peripheral blood samples using polymerase chain reaction (PCR).

**Results:**

Eight core PCD pathways were significantly associated with OM, mainly represented by apoptosis, autophagy, and non-classical forms such as cuproptosis and entosis. By integrating WGCNA with SHAP analysis, five hub genes (SORT1, KIF1B, TMEM106B, NPC1, and ATP6V0B) were identified as key diagnostic candidates. qPCR validation confirmed their significantly different expression between OM patients and healthy controls, supporting their utility as diagnostic biomarkers for early detection and treatment stratification.

**Conclusions:**

This study provides a comprehensive landscape of PCD involvement in OM, identifies novel diagnostic biomarkers, and highlights potential therapeutic targets for clinical intervention.

## 1 Background

OM is a debilitating infectious disease characterized by inflammation of the bone and bone marrow, affecting individuals of all ages worldwide (1, 2). With an estimated annual incidence of 21.8 cases per 100,000 person-years in the United States, OM poses a substantial healthcare burden, particularly among older adults and individuals with diabetes. Its pathogenesis is complex and multifactorial, with microbial infections—especially those caused by *Staphylococcus aureus*—being the primary contributors (3). Disease progression typically involves a cascade of pathological processes, including infection-induced immune responses, release of inflammatory cytokines, tissue necrosis, and disruption of bone remodeling (4).

Among the various biological mechanisms implicated in OM, PCD has emerged as a critical factor. As a tightly regulated process, PCD plays an essential role in maintaining tissue homeostasis and eliminating damaged or infected cells (5). It involves a series of orchestrated steps—sensing, activation, execution, and clearance—that ensure orderly and efficient cell death (6, 7). Since the concept of “apoptosis” was first introduced by Kerr, Wyllie, and Currie in 1972, research into PCD has expanded substantially, revealing multiple non-apoptotic forms of cell death, such as ferroptosis, pyroptosis, necroptosis, and parthanatos, which may also contribute to OM pathology (8, 9).

Recent studies have demonstrated that *Staphylococcus aureus* can manipulate host PCD pathways, such as necroptosis and pyroptosis pathways, to evade immune clearance and induce persistent bone tissue damage (10). Moreover, ferroptosis plays a critical role in the bone loss associated with chronic OM, which is driven primarily by iron release and oxidative stress (11). Emerging evidence suggests that ferroptosis-related genes possess significant diagnostic potential in this disease and are closely linked to patterns of immune infiltration (12). Furthermore, a diagnostic model based on autophagy- and immune-related genes has successfully identified two molecular subtypes characterized by distinct immune infiltration profiles, offering new insights into the pathogenesis and personalized immunotherapy of OM (13).

Despite these advances, the complex interactions among different PCD pathways in OM remain poorly understood. Elucidating the molecular crosstalk among apoptosis, necroptosis, pyroptosis, and ferroptosis is critical for developing new therapeutic strategies. Using high-throughput sequencing and WGCNA, researchers have identified several key regulatory molecules associated with OM. The present study aimed to establish a molecular signature based on core PCD-related molecules to predict disease severity, bone destruction, and inflammatory responses in OM patients. Our findings reveal the heterogeneity of OM patients and contribute to the assessment of clinical prognosis and the discovery of novel therapeutic targets.

## 2 Materials and methods

### 2.1 Data acquisition

**Figure 1:**
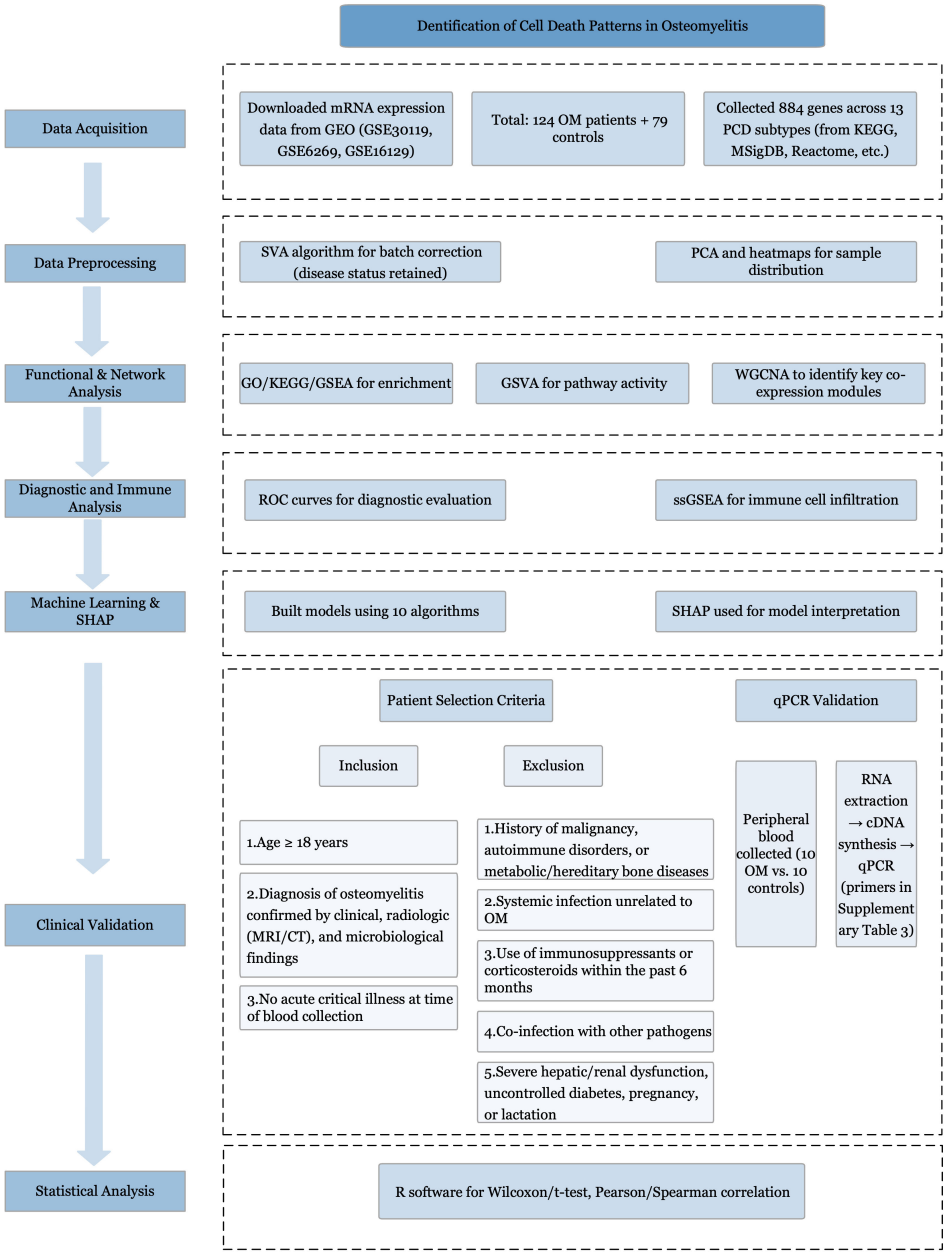
Study flowchart. Study flowchart illustrating the overall design and analytical procedures.

The primary datasets utilized in this study were derived from microarray-based mRNA expression profiles (GSE30119, GSE6269, and GSE16129) obtained from the GEO database (http://www.ncbi.nlm.nih.gov/geo/). The study cohort comprised peripheral blood samples from 124 patients diagnosed with OM and 79 healthy control subjects. The overall workflow is illustrated in **Figure 1**. The detailed characteristics and additional information regarding these datasets are summarized **Supplementary Table S1**.

The key regulatory genes associated with 13 distinct PCD modalities were curated from multiple sources, including the KEGG (14), GeneCards (15), Molecular Signatures Database (MSigDB) (16), Reactome (17), and relevant review articles (18). A comprehensive set of 884 genes was compiled, covering alkaliptosis (7 genes), apoptosis (136 genes), autophagy (151 genes), cuproptosis (14 genes), disulfidoptosis (4 genes), entosis (15 genes), ferroptosis (64 genes), lysosome-dependent cell death (255 genes), necroptosis (27 genes), NETosis (17 genes), oxeiptosis (26 genes), parthanatos (9 genes), and pyroptosis (27 genes). Clinical data from patients with OM were collected from the Department of Limb Orthopedics and Reconstructive Surgery, Second General Hospital of Fuzhou.

### 2.2 Data preprocessing

For the microarray-based mRNA expression profiles, batch effects were corrected via the surrogate variable analysis (SVA) algorithm (19). During SVA batch correction, the disease status (OM vs. control) was retained in the model as a biological covariate to avoid removing meaningful biological signals. Principal component analysis (PCA) and heatmap visualizations were subsequently performed to assess and illustrate the sample distribution patterns.

### 2.3 Pathway and functional enrichment analysis

Pathway and functional enrichment analyses were conducted via the R package “clusterProfiler” (20), including enrichment for KEGG pathways (21) and GO terms (22). In addition, gene set enrichment analysis (GSEA) (23) was performed to explore potential biological pathways. Terms with adjusted p values less than 0.05 were deemed statistically significant in the enrichment analysis.

### 2.4 Pathway activity calculation

GSVA, an unsupervised and non-parametric method, was applied to assess pathway-level changes in gene expression across individual samples. GSVA allows for quantification of gene set enrichment at the sample level. In our analysis, enrichment scores for each pathway were calculated independently, while allowing gene overlap among pathways. Potential crosstalk between PCD subtypes was considered during interpretation. GSVA was performed using the *GSVA* R package (24).

### 2.5 Construction of the coexpression network and identification of key modules via WGCNA

To identify PCD-related gene modules based on pathway enrichment scores from GSVA, we applied WGCNA (25). The *goodSamplesGenes* function was used to assess data quality, cluster samples, and eliminate outliers. To ensure scale-free topology, the optimal soft-thresholding power was determined using the *pickSoftThreshold* function. Gene modules were identified via the dynamic tree-cutting method, with the minimum module size set at 100 genes, a threshold supported by precedent in the literature (25).

### 2.6 Receiver operating characteristic curve analysis

ROC curve analysis was performed to evaluate the diagnostic efficacy of the 13 PCD pathways and core death-related genes (26).

### 2.7 Analysis of immune cell proportions

We employed single-sample gene set enrichment analysis (ssGSEA) to estimate immune cell proportions. Feature gene panels for immune cell types were obtained from published sources (27, 28). The *GSVA* R package (24) was used to convert the expression matrix of individual genes into an immune cell-type score matrix.

### 2.8 Enhancing model interpretability with SHAP

Multiple machine learning algorithms were evaluated, including partial least squares (PLS), random forest (RF), decision tree (DT), support vector machine (SVM), logistic regression, k-nearest neighbors (KNN), extreme gradient boosting (XGBoost), gradient boosting machine (GBM), neural network, and generalized linear model boosting (glmBoost). To enhance interpretability, we used SHAP, which assigns each feature a contribution value to the model output.

### 2.9 Clinical sample collection and PCR validation

#### 2.9.1 Patient recruitment and eligibility criteria

Adult patients (≥18 years) with clinically, radiologically, and microbiologically confirmed OM were recruited from the Department of Limb Orthopedics and Reconstructive Surgery, Second General Hospital of Fuzhou. Diagnosis was established by clinical manifestations, magnetic resonance imaging (MRI) or computed tomography (CT), and positive microbiological cultures. Written informed consent was obtained.

Inclusion criteria: (1) age ≥18 years; (2) clinically, radiologically, and microbiologically confirmed OM; (3) no acute critical illness.

Exclusion criteria: (1) malignancy, autoimmune disorders, or metabolic/hereditary bone disease; (2) acute systemic infection unrelated to OM; (3) immunosuppressive therapy or systemic corticosteroid use within 6 months; (4) co-infection with other infectious diseases; (5) severe hepatic/renal dysfunction, poorly controlled diabetes, pregnancy, or lactation.

#### 2.9.2 Sample collection and RNA extraction

Peripheral blood (2 mL) was collected into EDTA tubes from 10 OM patients and 10 matched healthy controls. Total RNA was extracted using TRIzol reagent (Invitrogen, USA) per manufacturer’s instructions. RNA concentration and purity (A260/A280) were measured with a NanoDrop 2000 spectrophotometer (Thermo Fisher Scientific, USA), ensuring values of 1.8–2.0.

#### 2.9.3 cDNA synthesis and qPCR

One microgram of total RNA was reverse transcribed using the PrimeScript RT reagent kit (Takara Bio, Japan). Quantitative PCR was performed with Taq PCR Master Mix (Tiangen Biotech, China) on a Bio-Rad T100 thermal cycler. GAPDH served as an internal control. Primer sequences are in **Supplementary Table S3**. Thermocycling: 95°C 5 min, 40 cycles of 95°C 10 s, 60°C 30 s, 72°C 30 s, followed by melt curve analysis. PCR products were verified by 2% agarose gel electrophoresis (Invitrogen) and visualized on a Gel Doc XR+ system (Bio-Rad, USA).

#### 2.9.4 Laboratory equipment

Key instruments included: high-speed refrigerated centrifuge (H1850R, Xiangyi, China), ultra-low temperature freezer (DW-86L500, AUCMA, China), water bath (DK-8D, Jinghong, China), biological safety cabinet (HF1200LC, Likang, China), analytical balance (NBL-214e, AE ADAM, UK), and ultrapure water system (WP-UP-YJ-10, Watepur, China).

### 2.10 Statistical analysis

Statistical analyses were performed using R software (version 4.2.1; https://www.r-project.org/). For two-group comparisons, either the Wilcoxon rank-sum test or Student’s *t* test was applied. Correlation analyses were conducted via Pearson’s or Spearman’s method, depending on data type. All tests were two-tailed, with p <0.05 considered statistically significant.

## 3 Results

### 3.1 Dysregulated gene expression highlights enhanced immune and inflammatory pathways in OM

To ensure comparability across datasets, three OM-related transcriptome datasets were merged and normalized after batch effect removal using the SVA algorithm. PCA and box plots confirmed the effective elimination of batch effects (**Supplementary Figure S1a–d**).

**Figure 2:**
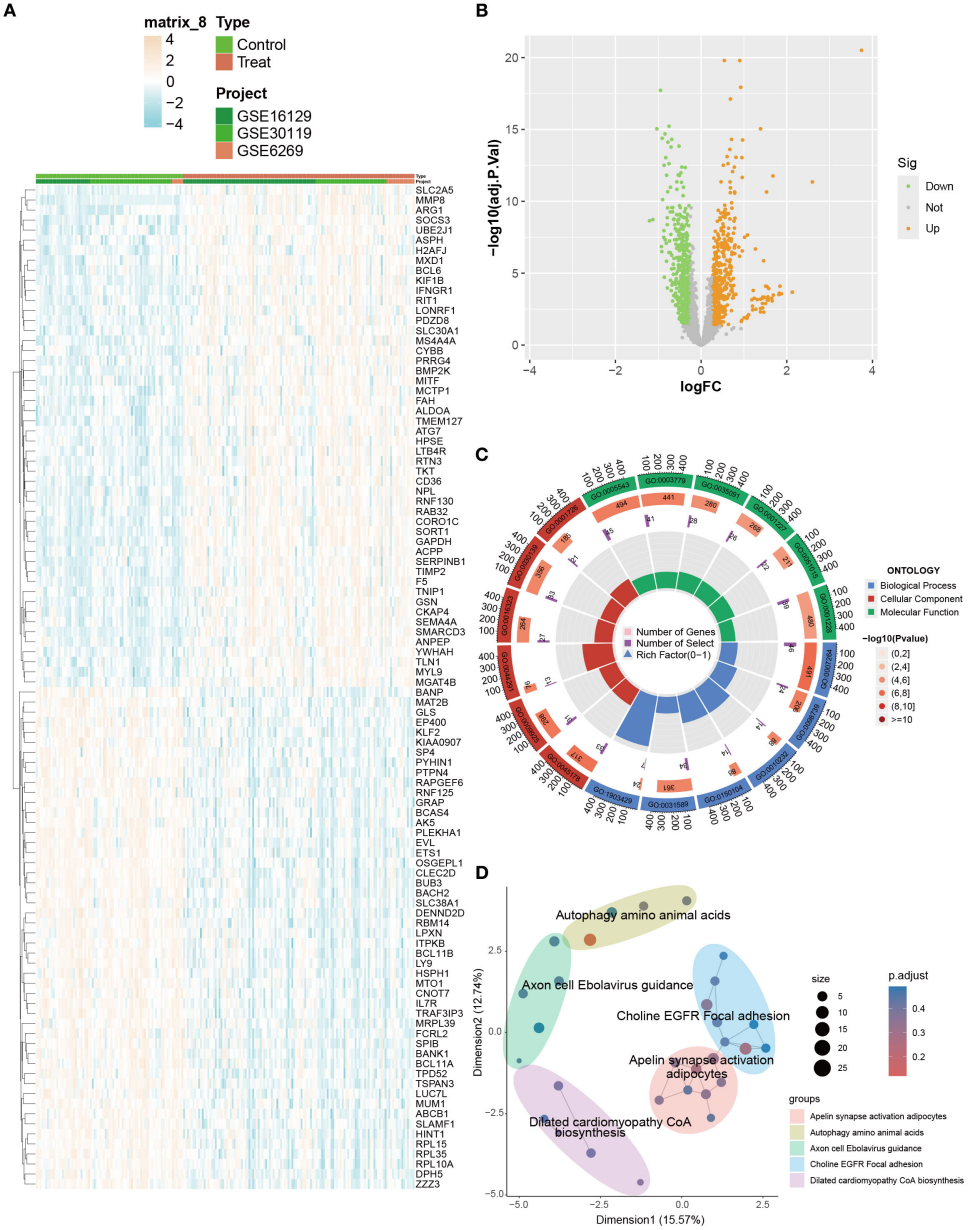
Identification and enrichment analysis of DEGs between OM and control groups. **(A)** Heatmap visualizing the expression patterns of DEGs across samples. **(B)** Volcano plot displaying significantly up- and downregulated DEGs (|logFC| > 0.585). **(C, D)** GO and KEGG enrichment analyses highlighting biological processes and pathways associated with immune and inflammatory responses.

Differential expression analysis identified 915 DEGs between OM and control groups (p < 0.05, |logFC| > 0.585), with 450 upregulated and 465 downregulated genes (**Figures 2A, B**). GO enrichment highlighted processes associated with cytoskeletal organization (e.g., small GTPase-mediated signaling), cell adhesion and migration (e.g., vascular transport, focal adhesion), and signaling regulation (e.g., phospholipid binding, actin binding) (**Figure 2C**).

KEGG analysis further revealed enrichment in immune- and infection-related pathways (e.g., HTLV-1 infection, tuberculosis, toxoplasmosis), adhesion signaling (focal adhesion), and metabolic regulation (apelin signaling, amino acid biosynthesis) (**Figure 2D**). Collectively, these results indicate that OM is characterized by dysregulated gene expression, accompanied by enhanced immune and inflammatory responses, abnormal adhesion, and cytoskeletal remodeling, which collectively contribute to disease progression.

### 3.2 PCD-related transcriptional alterations reveal enhanced immune regulation and distinct pathway activity in OM

**Figure 3:**
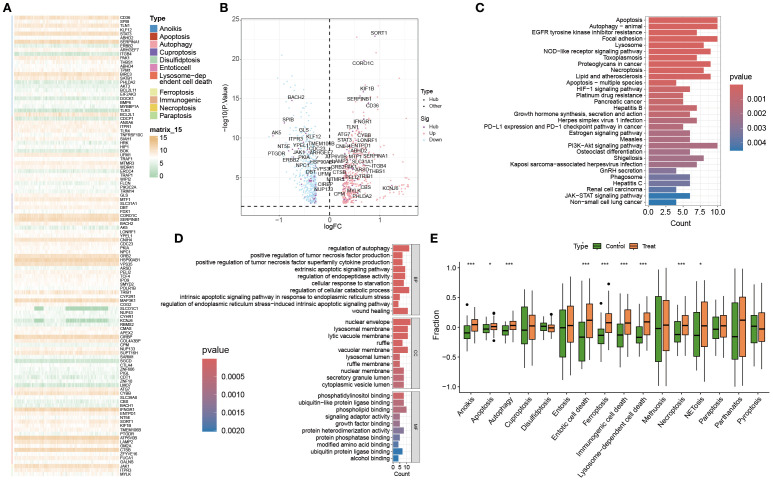
Analysis of PCD-related gene expression and pathway activity in the control and OM groups. **(A, B)** Heatmap and volcano plot showing differentially expressed PCD-related genes between the control and OM groups. **(C, D)** GO and KEGG pathway enrichment analyses of these DEGs. **(E)** Box plots illustrating the GSVA scores of 13 PCD pathways in both groups. *, **, and *** indicate p < 0.05, p < 0.01, and p < 0.001, respectively.

We next evaluated differences in PCD-related gene expression between OM and control samples. The heatmap and volcano plot (**Figures 3A, B**) demonstrated widespread transcriptional dysregulation across multiple PCD subtypes, including apoptosis, autophagy, entosis, ferroptosis, lysosome-dependent cell death, necroptosis, and pyroptosis.

GO enrichment highlighted key biological processes such as regulation of autophagy, positive regulation of TNF production, and activation of extrinsic apoptotic signaling (**Figure 3C**). These genes were primarily involved in protein binding, phosphatidylinositol interaction, and ubiquitin-like protein ligase activity—molecular functions central to cell death control.

KEGG pathway analysis further revealed enrichment in canonical immune and survival signaling pathways, most notably the NOD-like receptor and PI3K-Akt cascades (**Figure 3D**), underscoring their pivotal role in immune regulation and PCD execution.

To systematically quantify PCD activity, GSVA was applied to 13 PCD-related pathways (**Figure 3E**). Seven pathways—including apoptosis, autophagy, immunogenic cell death, ferroptosis, necroptosis, NETosis, and pyroptosis—were significantly upregulated in OM, whereas entosis, alkaliptosis, and parthanatos were downregulated. These enrichment profiles revealed not only pathway-specific alterations but also potential cross-talk among distinct PCD programs, highlighting a coordinated reprogramming of cell death mechanisms in OM.

### 3.3 Immune cell infiltration is markedly elevated in OM and tightly associated with dysregulated PCD pathways

**Figure 4:**
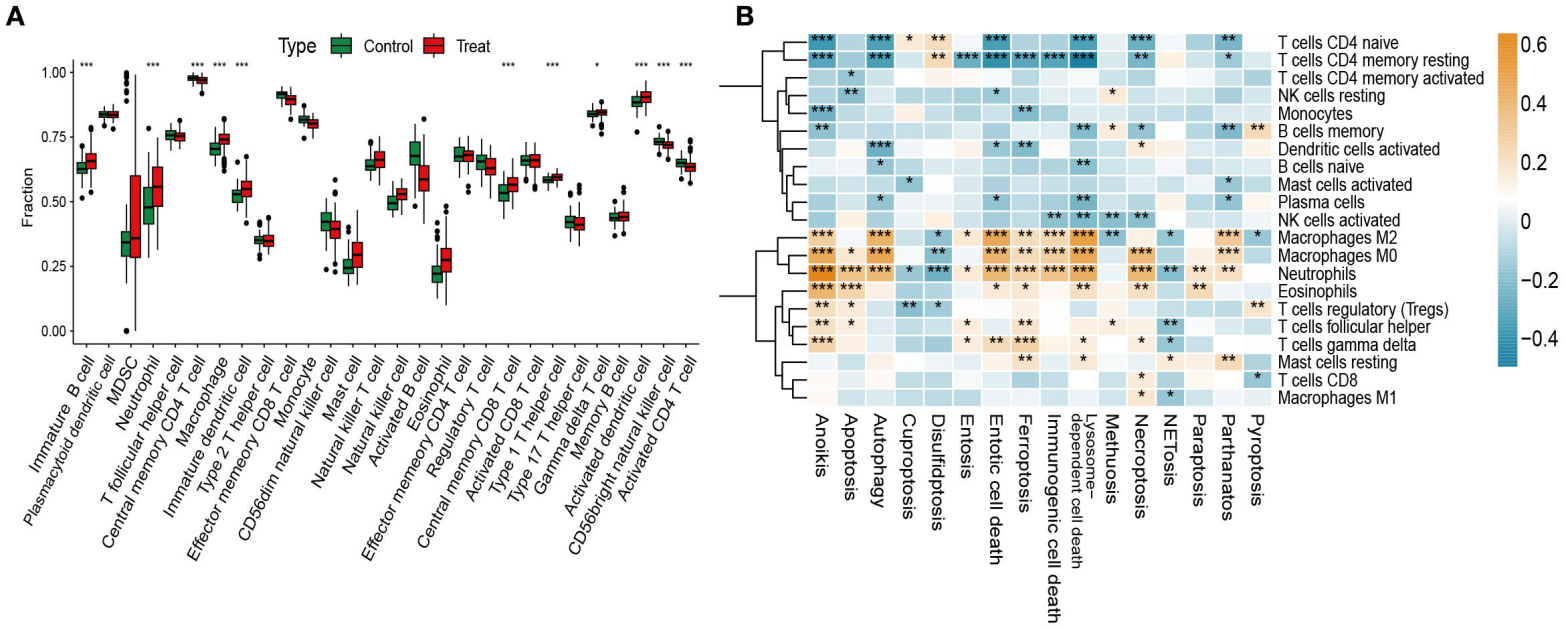
Immune infiltration analysis and its correlation with PCD pathway activity. **(A)** Immune cell scores estimated via ssGSEA across both groups. **(B)** Correlations between the GSVA scores of 13 PCD pathways and immune cell subsets. *p < 0.05, **p < 0.01, ***p < 0.001.

Stimulus-induced cell death can activate the immune system and elicit responses against dead-cell antigens (29). Immune cell infiltration is therefore a key driver of OM initiation and progression (30). Using ssGSEA, we observed significantly higher infiltration of T cells, macrophages, dendritic cells, and eosinophils in OM compared with controls (**Figure 4A**).

We next analyzed correlations between GSVA-derived PCD pathway activity and immune infiltration. Apoptosis, necroptosis, immunogenic cell death, and ferroptosis showed strong positive associations with activated immune subsets, particularly CD4^+^/CD8^+^ T cells and M1/M2 macrophages, suggesting their role in enhancing immune responses through cell recruitment. In contrast, autophagy and lysosome-dependent cell death were negatively correlated with regulatory T cells (Tregs) and M0 macrophages, implying suppression of immune activation (**Figure 4B**).

Together, these findings highlight the intricate interplay between dysregulated PCD and immune infiltration in OM, supporting the view that aberrant cell death processes actively shape the inflammatory microenvironment.

### 3.4 Clinical relevance of autophagy, lysosome-dependent, and entotic cell death pathways in OM

**Figure 5:**
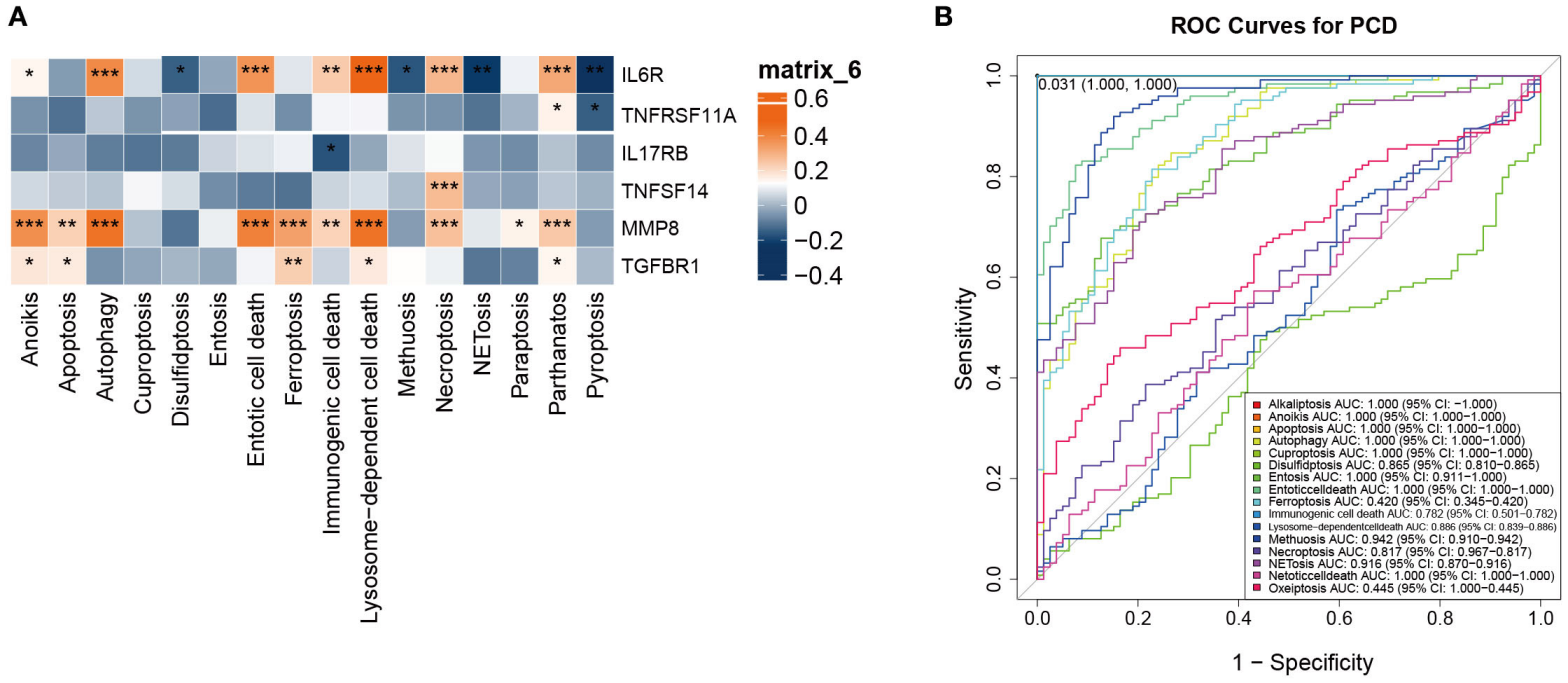
Identification of the clinical significance of the 13 types of PCD in OM. **(A)** Correlation analysis between the 13 PCD-related GSVA scores and bone marrow inflammation markers in OM. **(B)** ROC curve analysis of the 13 PCD pathways in OM. *p < 0.05, **p < 0.01, ***p < 0.001. ns: no statistically significant difference.

We next investigated the clinical significance of PCD pathways by correlating their GSVA scores with representative inflammatory biomarkers of osteomyelitis, including IL6R, MMP8, TNFRSF11A, IL17RB, TNFSF14, and TGFBR1 (**Figure 5A**). Pearson correlation heatmaps revealed that IL6R and MMP8 expression exhibited the strongest positive associations with autophagy, lysosome-dependent cell death, and entosis (p < 0.001). Here, “clinical correlation” refers to statistically significant relationships between pathway activity and clinically recognized inflammatory mediators, implying that these death modalities may drive inflammatory amplification and tissue destruction in OM.

Notably, IL6R, a pivotal receptor mediating immune-inflammatory signaling, reflects immune activation status, while MMP8, a neutrophil-derived metalloproteinase, is involved in extracellular matrix degradation and acute inflammation. Both markers serve as clinically relevant indicators of infection activity and tissue injury, underscoring their value in evaluating OM pathophysiology.

To further assess their diagnostic potential, ROC curve analysis was performed. Autophagy, lysosome-dependent cell death, and entosis all achieved high AUC values in distinguishing OM patients from controls (**Figure 5B**). These findings highlight that beyond their mechanistic links to inflammation, these PCD pathways may also serve as candidate biomarkers for early diagnosis.

Collectively, autophagy, lysosome-dependent cell death, and entosis represent the most clinically relevant PCD pathways in OM, offering promising avenues for future work on inflammation modulation and diagnostic biomarker development.

### 3.5 Anoikis- and lysosome-dependent modules drive immune and inflammatory networks in OM

**Figure 6:**
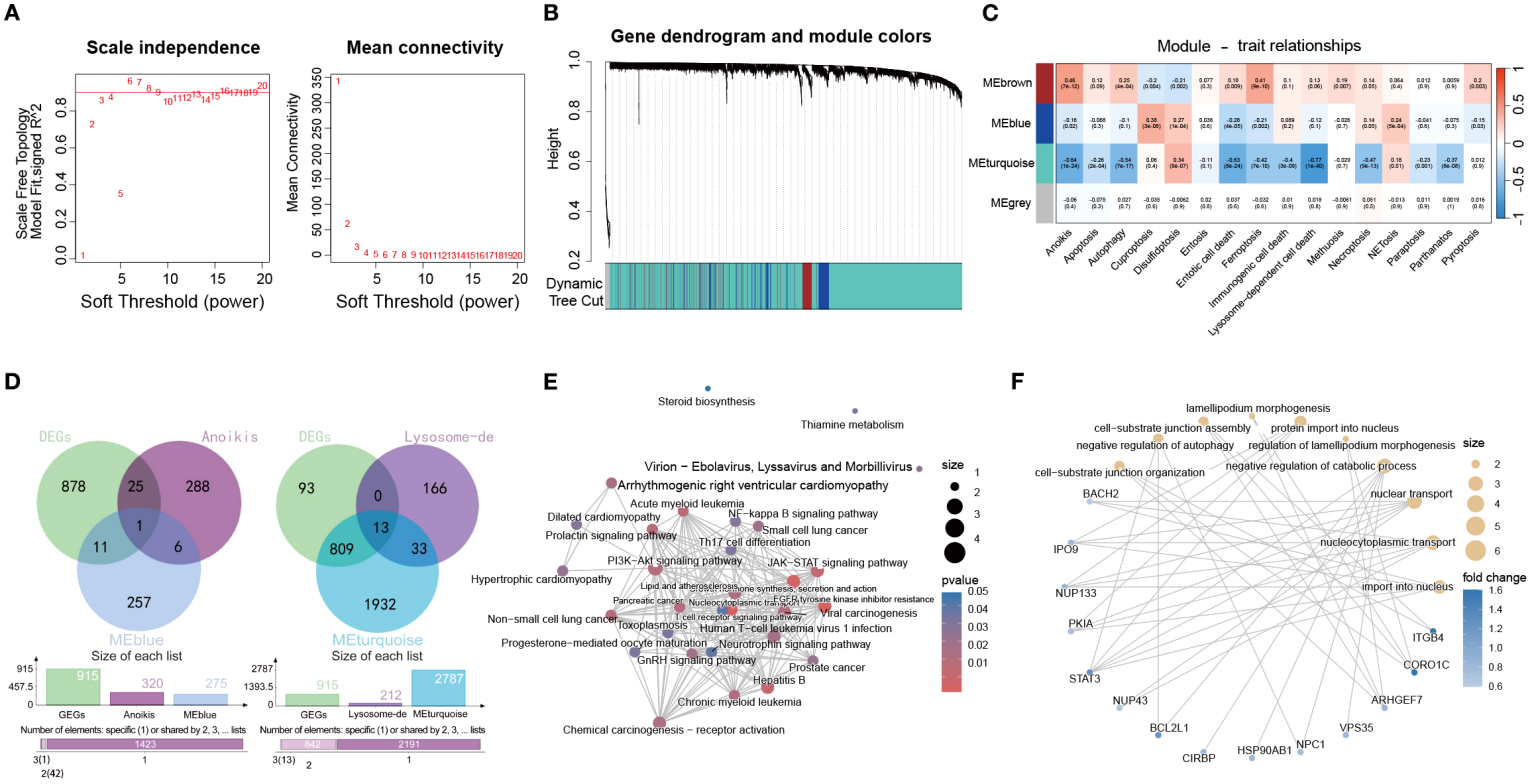
Identification of PCD-associated gene modules in the OM via WGCNA. **(A)** Analysis of scale-free topology and average connectivity across various soft thresholds. **(B)** Gene cluster dendrogram, with colors indicating distinct coexpression modules. **(C)** Heatmap displaying the correlation between module eigengenes and GSVA scores of 13 PCD pathways. **(D)** Venn diagrams illustrating overlaps between blue or turquoise module genes and DEGs from the control and OM groups. **(E, F)** Functional enrichment (KEGG and GO) of overlapping gene sets.

To identify modules most relevant to PCD in OM, we performed WGCNA based on GSVA enrichment scores of 13 pathways. A soft-thresholding power of 8 was selected to ensure scale-free topology (**Figure 6A**). Hierarchical clustering identified the MEblue module as most strongly correlated with anoikis, while the MEturquoise module showed the highest correlation with lysosome-dependent cell death (**Figures 6B, C**).

We then intersected genes from these modules with DEGs between OM and controls, yielding module-specific sets (**Figure 6D**). KEGG enrichment (**Figure 6E**) highlighted PI3K–Akt and JAK–STAT signaling, while GO analysis (**Figure 6F**) pointed to nucleocytoplasmic transport and autophagy regulation, emphasizing their role in immune control and cell fate.

Together, these results indicate that anoikis- and lysosome-dependent modules are closely linked to immune regulation and inflammation in OM, representing potential therapeutic targets.

### 3.6 Identification of key diagnostic genes via SHAP-based machine learning analysis

**Figure 7:**
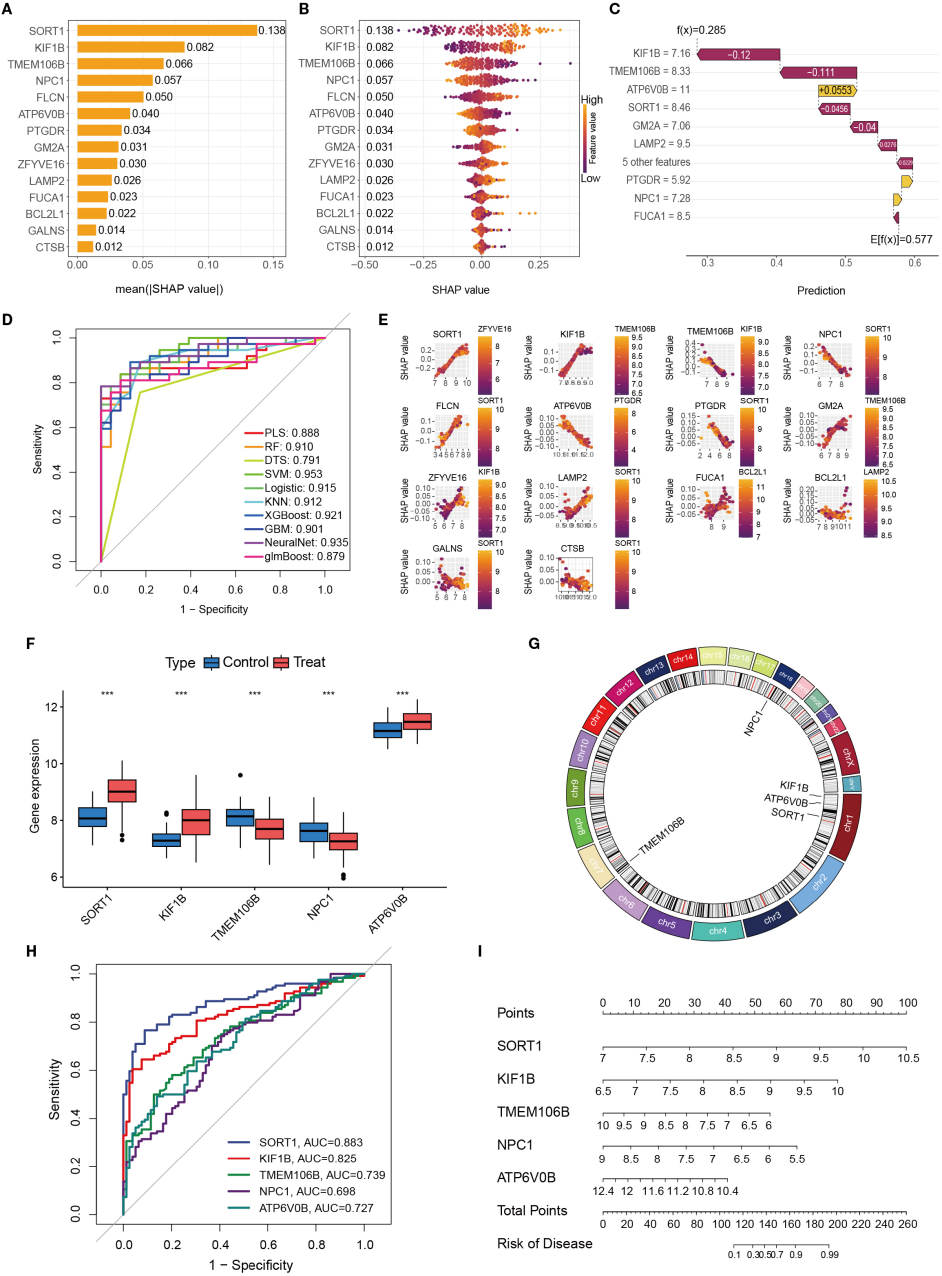
The importance and contribution of each characteristic variable in the ML model were evaluated via Shap analysis. **(A)**. A feature importance ranking graph that can be interpreted on the basis of SHAP. **(B)** Feature colony map interpretable via SHAP; **(C)**. Interpretable feature variable waterfall diagram based on SHAP; **(D)**. Contribution maps of individual features interpretable via SHAP. **(E)**. Performance comparison of different machine learning models in OM prediction (ROC curves). **(F)**. Differential expression of key genes in disease models. **(G)** Chromosomal gene distribution of key genes **(H)**. ROC curve analysis for key genetic biomarkers. **(I)** Genetic risk prediction nomogram. *, **, and *** indicate p < 0.05, p < 0.01, and p < 0.001, respectively.

To comprehensively evaluate the diagnostic contribution of candidate genes in OM, we applied SHAP-based machine learning analysis across multiple models. Fourteen genes were assessed, and SHAP values were used to quantify their influence on model predictions. Higher SHAP values indicate stronger discriminatory ability between OM patients and healthy controls (**Figures 7A–E**).

Across models, SORT1, KIF1B, TMEM106B, NPC1, and ATP6V0B consistently ranked among the top contributors, highlighting their potential diagnostic value. We further validated these genes by examining their expression profiles in GEO datasets, determining chromosomal locations, and conducting ROC curve and nomogram analyses (**Figures 7F–I**). Among them, SORT1 demonstrated the highest and most stable diagnostic performance, with consistently superior AUC values across analyses, underscoring its robustness as a biomarker.

Taken together, these results highlight SORT1, KIF1B, TMEM106B, NPC1, and ATP6V0B as promising diagnostic biomarkers for OM, with SORT1 emerging as the most compelling candidate for future clinical translation.

### 3.7 PCR-based validation of five gene expression biomarkers

**Figure 8:**
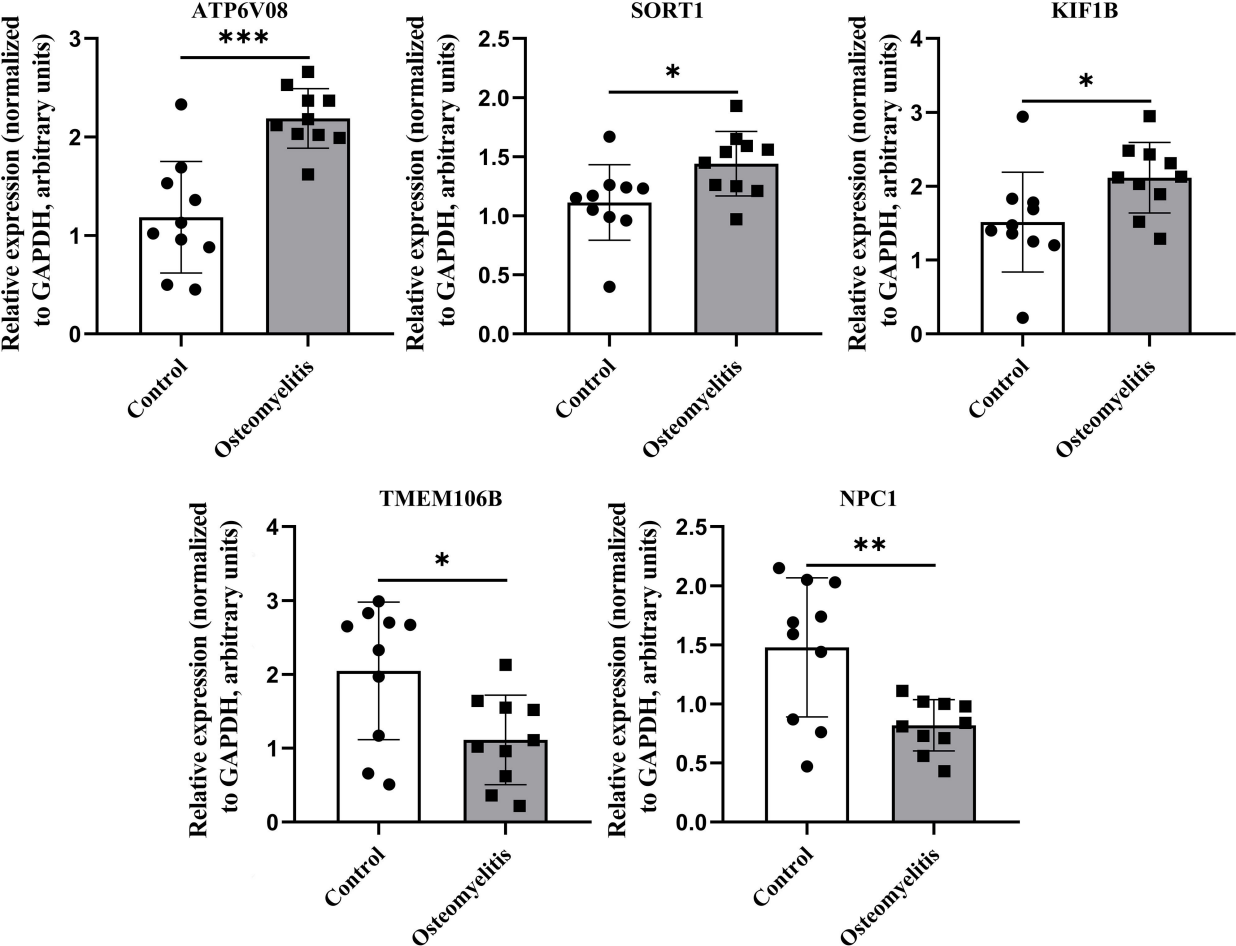
Differences in the expression of key genes between patients with OM and healthy controls. Relative expression levels of key genes in peripheral blood from OM patients (n = 10) and healthy controls (n = 10). GAPDH served as internal reference. *, **, and *** indicate p < 0.05, p < 0.01, and p < 0.001, respectively.

Peripheral blood samples from patients with OM and healthy controls were collected for PCR validation of the five key genes (SORT1, KIF1B, TMEM106B, NPC1, and ATP6V0B) identified through integrated bioinformatics and SHAP analyses. All OM cases were recruited from the Department of Limb Orthopedics and Reconstructive Surgery, Fuzhou Second Hospital, and only patients meeting the inclusion criteria without concurrent infectious or severe systemic diseases were enrolled. PCR results revealed significant expression differences between the OM and control groups, with SORT1 showing the most pronounced upregulation in OM (**Figure 8**). These experimental findings not only corroborate the computational predictions but also highlight the potential of these genes—particularly SORT1—as clinically relevant biomarkers for OM diagnosis and possible targets for mechanistic investigation.

## 4 Discussion

In this study, we systematically profiled the activity of 13 PCD pathways in OM and elucidated their associations with PCD-related gene expression and inflammatory mediators. Using microarray-based transcriptomic data, we developed a SHAP-interpretable machine learning model, which identified five core PCD-related genes—SORT1, KIF1B, TMEM106B, NPC1, and ATP6V0B—as potential diagnostic biomarkers. These findings provide novel insights into the molecular interplay between cell death modalities and inflammatory responses in OM.

Apoptosis is a highly programmed form of cell death that is primarily mediated by the caspase family of serine proteases and is widely regarded as a self-executed “suicide” mechanism of the cell (31). In recent years, studies have shown that apoptosis plays an important role in the onset and progression of OM, where dysregulated apoptosis—particularly excessive loss of bone-forming and immune cells—has been implicated in both structural bone damage and compromised local immunity (32).

SORT1, a member of the Vps10p receptor family, is known to modulate apoptosis in neuronal and immune contexts (33, 34). In bone-related diseases, SORT1 promotes cell death by mediating apoptotic signals dependent on neurotrophic factors, such as proNGF, and may exacerbate bone tissue damage and resorption following infection (35). Although there are currently no direct studies investigating the role of SORT1 in OM, previous research has indicated that the upregulation of SORT1 can enhance cell death and tissue damage in chronic inflammatory environments (36).

On the other hand, kinesin family member 1B (KIF1B), a microtubule-associated motor protein, participates in organelle transport and exerts pro-apoptotic effects under cellular stress (37). It has been reported that loss of the KIF1B-β isoform can enable tumor cells to evade apoptosis, suggesting that in the context of OM, dysfunction of KIF1B may impair the response of local immune and bone cells to infection, thereby promoting infection spread and bone destruction (38). However, direct research on the associations among SORT1, KIF1B, and apoptosis in OM remains limited, and further investigations are needed to elucidate the underlying mechanisms involved.

Autophagy—another essential PCD modality—maintains cellular homeostasis by lysosomal degradation of damaged organelles and misfolded proteins (39). During infection, autophagy plays a critical role in regulating immune responses and facilitating pathogen clearance. TMEM106B, a lysosomal transmembrane protein, is a critical regulator of lysosomal activity and autophagic flux (40). Studies have shown that the overexpression of TMEM106B can impair lysosomal trafficking and autophagy, leading to protein aggregation and cellular dysfunction (41, 42). In our study, we observed significant alterations in autophagy-related pathways in OM tissues. Evidence suggests that impaired autophagy can promote chronic infection persistence by enhancing cellular stress and tissue damage (43). It is hypothesized that TMEM106B dysregulation may similarly limit intracellular pathogen clearance during OM, thereby exacerbating infection spread and bone tissue destruction. Targeting TMEM106B-mediated autophagy dysfunction may offer a novel therapeutic strategy to improve pathogen clearance and reduce bone damage in patients with OM.

Lysosome-dependent cell death (LDCD) arises from increased lysosomal membrane permeability, releasing proteolytic enzymes into the cytosol and initiating irreversible cellular degradation (44). This process results in the leakage of lysosomal contents into the cytoplasm, the activation of degradative enzymes, and the subsequent destruction of cellular structures, ultimately leading to cell death (45). LDCD plays a critical role in maintaining cellular homeostasis and regulating inflammatory responses, and dysfunction of lysosomal function can contribute to the development and progression of various diseases (46).

NPC1, a pivotal mediator of cholesterol trafficking and lysosomal membrane stability (47). Studies have shown that loss of NPC1 function can lead to lipid accumulation within lysosomes, membrane rupture, and the initiation of LDCD (48). Although direct evidence linking NPC1 to OM is currently lacking, disturbances in cholesterol homeostasis are closely associated with abnormal immune responses and enhanced tissue destruction, suggesting that NPC1-mediated LDCD may play a potential role in the progression of OM.

ATP6V0B, a subunit of the vacuolar-type H+-ATPase (V-ATPase) complex, is responsible for maintaining lysosomal acidification and degradative capacity (49). Research has indicated that ATP6V0B dysfunction impairs lysosomal enzymatic activity, leads to the accumulation of damaged organelles, and subsequently triggers lysosome-dependent cell death (50). Given lysosomal dependency for osteoclast survival and bone resorption, ATP6V0B dysregulation in OM could amplify bone destruction and chronic inflammation.

Collectively, these findings suggest that SORT1, KIF1B, TMEM106B, NPC1, and ATP6V0B represent distinct molecular nodes within the full spectrum of PCD pathways in OM, where their dysregulation may synergistically drive bone destruction, chronic inflammation, and impaired pathogen clearance.

This study also presents several limitations. First, although we preliminarily validated the abnormal expression of genes associated with apoptosis, autophagy, and LDCD in clinical samples from patients with OM, functional assays that directly elucidate the mechanistic roles of these genes in disease progression were not performed. Further *in vitro* and *in vivo* experiments are warranted to systematically investigate the contributions of LDCD and its related genes to the pathogenesis of OM. Such efforts will deepen the understanding of disease mechanisms and provide a theoretical foundation for the development of novel therapeutic strategies.

Second, the clinical validation was based on a relatively limited sample size. The diagnostic risk model constructed in this study relies on selected gene signatures, which may not comprehensively capture all key regulatory components implicated in OM. Additional studies involving larger and more diverse patient cohorts are needed to confirm the robustness and generalizability of the findings.

Third, the pathogenesis of OM is likely modulated not only by transcriptional alterations but also by epigenetic mechanisms, including DNA methylation and histone modifications. Future research integrating epigenomic profiling may enhance the accuracy and depth of biological interpretation, and facilitate the identification of more reliable diagnostic biomarkers and potential therapeutic targets.

## 5 Conclusions

In summary, this study identified eight core PCD pathways implicated in OM, including apoptosis, autophagy, and nonclassical forms such as cuproptosis and entosis, which aligns with the results presented. By integrating WGCNA with SHAP-based machine learning, we identified five key diagnostic genes: SORT1, KIF1B, TMEM106B, NPC1, and ATP6V0B. Validation using peripheral blood samples confirmed significant differential expression of these genes, underscoring their potential diagnostic value in clinical settings.

Collectively, these findings provide novel insights into the molecular mechanisms underlying OM and highlight promising targets for clinical assessment and therapeutic intervention.

## Data Availability

The datasets presented in this study can be found in online repositories. The names of the repository/repositories and accession number(s) can be found in the article/**Supplementary Material**.
