# Correction: Identification and analysis of diverse cell death patterns in osteomyelitis via microarray-based transcriptome profiling and clinical data

**DOI:** 10.3389/fimmu.2025.1721889

**Published:** 2025-10-20

**Authors:** Tianxuan Feng, Peisheng Chen, Fengfei Lin

**Affiliations:** ^1^ Fujian University of Traditional Chinese Medicine, Fuzhou, Fujian, China; ^2^ Department of Orthopedic, Fuzhou Second General Hospital, School of Clinical Medicine of Fujian Medical University, Fujian Provincial Clinical Medical Research Center for First Aid and Rehabilitation in Orthopedic Trauma, Fuzhou, Fujian, China

**Keywords:** osteomyelitis, programmed cell death, transcriptome profiling, biomarkers, machine learning

An incorrect **Funding** statement was provided. The correct **Funding** statement reads:

“The author(s) declare that financial support was received for the research and/or publication of this article. This work was supported by grants from the Center for First Aid and Rehabilitation in Orthopedic Trauma (2020Y2014), the Science and Technology Planning Project of Fuzhou (2022-R-009), and the Fujian Provincial Natural Science Foundation (Grant No. 2025J011352).”

There was an error in the second affiliation. The country name “China” was inadvertently placed at the beginning of the affiliation. The correct affiliation is:

“Department of Orthopedic, Fuzhou Second General Hospital, School of Clinical Medicine of Fujian Medical University, Fujian Provincial Clinical Medical Research Center for First Aid and Rehabilitation in Orthopedic Trauma, Fuzhou, Fujian, China.”

The original version of this article has been updated.

