# Supplementary material for: Identification and analysis of diverse cell death patterns in osteomyelitis via microarray-based transcriptome profiling and clinical data
Source: Front Immunol. 2025 Sep 19;16:1630172. doi: 10.3389/fimmu.2025.1630172 (PMC12491029; doi:10.3389/fimmu.2025.1630172)
Supplement: Supplementary file 1 [file SupplementaryFile1.zip › Suppl. Image 1.DOCX]

Supplementary Fig 1

a

b

c

d
